# Harnessing miRNA dynamics in HIV-1-infected macrophages: Unveiling new targeted therapeutics using systems biology

**DOI:** 10.1016/j.csbj.2025.04.040

**Published:** 2025-05-01

**Authors:** R. Harshithkumar, Mollina Kaul, Madhuri Chandane-Tak, Nikhat J. Siddiqi, Abdul Malik, Abdul Arif Khan, Anupam Mukherjee

**Affiliations:** a ICMR-National Institute of Translational Virology and AIDS Research, Pune, MH, India; b King Saud University, Riyadh, Saudi Arabia; c AcSIR - Academy of Scientific & Innovative Research, Ghaziabad, UP, India; d ICMR-National Institute of Virology, Pune, MH, India

**Keywords:** HIV-1, MiRNA, Microarray, Gene ontology, Biological networks, Molecular function, Pathogenesis

## Abstract

**Background:**

The interaction between HIV-1 and host immune cells, particularly macrophages, is crucial in understanding viral persistence and pathogenesis. This study aims to explore the impact of HIV-1 infection on macrophage microRNA (miRNA) expression profiles using a systems biology approach to uncover the potential role of miRNAs in modulating macrophage functionality and identify key miRNA targets that may serve as therapeutic avenues.

**Methods:**

PMA-differentiated THP-1 cells were used to model macrophage infection with HIV-1. A custom miRNA microarray was performed to identify dysregulated miRNAs following infection. miRTarBase was utilized for miRNA target identification, revealing gene targets associated with the dysregulated miRNAs. A protein-protein interaction (PPI) map of miRNA targets and their first interactors was constructed, with key nodes identified based on a calculated disease score, which considered degree, betweenness centrality, average shortest path length, and clustering coefficient. Gene Ontology molecular function analysis was also conducted on the identified targets.

**Results:**

The miRNA microarray identified 23 dysregulated miRNAs in HIV-1-infected macrophages, with 8 upregulated and 15 downregulated. Among these, the top 10 dysregulated miRNAs targeted over 2000 unique genes. PPI analysis revealed key nodes in the upregulated miRNA network, including *APP*, *MYC*, *ESR2*, *RAF1*, and *HIST1H4A*, while *ZRANB1*, *HSPA8*, *TGOLN2*, *HSPA5*, and *BRD4* were prominent in the downregulated miRNA network. Notably, *KRAS*, *CUL3*, *TP53*, *ESR1*, and *PARP1* were influenced by both upregulated and downregulated miRNAs. Gene Ontology analysis indicated that the targeted genes were involved in processes such as protein and RNA binding, ATPase activity, and ribosomal function.

**Conclusions:**

HIV-1 infection induces significant dysregulation of miRNAs in macrophages, impacting a wide array of gene targets and molecular functions. These findings suggest that miRNA-mediated regulation may play a crucial role in HIV-1 pathogenesis within macrophages and present potential targets for miRNA-based therapeutic strategies.

## Graphical Abstract

**Figure:**
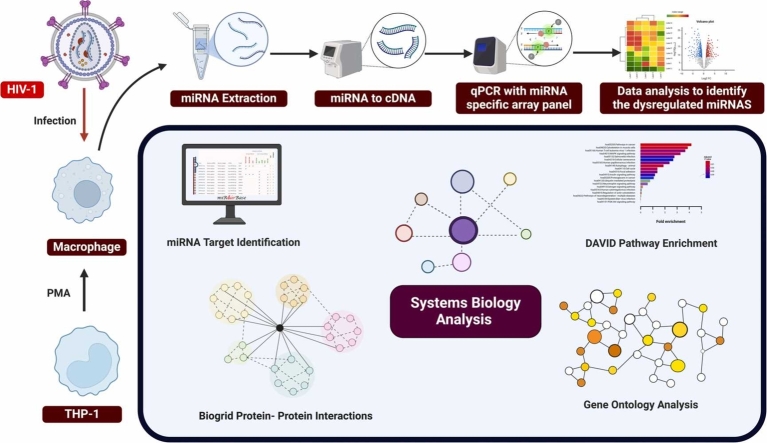

## 1 Introduction

HIV-1 infection remains a significant global health challenge, affecting approximately 38 million people worldwide despite advances in antiretroviral therapy (ART) [1], [2], [3], [4]. While current treatments have transformed HIV from a fatal disease to a manageable chronic condition, ART cannot eliminate the virus completely from infected individuals [5], [6]. This is largely due to the virus's ability to establish persistent reservoirs in host cells, particularly macrophages and resting CD4^+^ T cells, which evade immune detection and drug targeting. These reservoirs contain integrated proviral DNA that can reactivate upon treatment interruption, necessitating lifelong therapy [7], [8], [9]. The economic burden of continuous treatment, emergence of drug resistance, and long-term side effects of ART further highlight the urgent need for novel therapeutic approaches [10], [11]. Understanding the molecular mechanisms underlying HIV-1 pathogenesis, latency establishment, and viral persistence is therefore critical for developing strategies aimed at HIV remission or cure [12], [13].

The human genome was once thought to be a blueprint, all of which contained instructions solely dedicated to coding proteins. However, the Human Genome Project and subsequent findings revealed that only a tiny fraction of our 3 billion base pairs, fewer than 20,000 genes and encode proteins [14], [15], [16]. This surprising revelation underscored the complexity of genetic regulation, highlighting the roles of non-coding regions in gene expression and cellular function. Among these non-coding elements, microRNAs (miRNAs) have emerged as critical regulators of gene expression, influencing an array of biological processes and disease mechanisms [17]. MicroRNAs are small, non-coding RNA molecules, typically 21–25 nucleotides in length, that function by binding to complementary sequences on target mRNAs, leading to translational repression or mRNA degradation [18], [19]. This post-transcriptional regulation enables miRNAs to fine-tune the expression of numerous genes, thereby orchestrating complex cellular processes and maintaining homeostasis [20], [21]. Recent studies have signified the role of miRNAs in immune cell function and differentiation [22], [23], [24], [25], [26]. Macrophages, key players in the innate immune response, are highly plastic cells capable of adopting various functional states in response to microenvironmental signals [27], [28], [29]. This plasticity is crucial for their roles in pathogen inflammatory response and clearance, with miRNAs playing a notable role in regulating these macrophage functions [30], [31], [32]. However, various pathological conditions, including cancer, neurodegenerative disease and viral infections, are associated with dysregulation of microRNAs in various cell types, with macrophages being no exception [33], [34], [35], [36], [37], [38], [39], [40], [41]. In particular, viral infection of macrophages can be little enigmas as they employ dexterous strategies to evade host immune responses and the miRNA dysregulation can be attributed to both virus-induced modifications of the cellular environment and host antiviral defences [42], [43], [44], [45].

The relationship between microRNAs and viral infections is particularly relevant to viral pathogenesis, as microRNAs are widely recognized as key regulators of gene expression in macrophages during numerous viral infections [46]. Specifically in HIV-1 infection, recent studies have sparingly established that miRNAs play a crucial role in the immunopathogenesis by modulating immune activation, inflammatory pathways, and viral control [47], [48], [49]. Specific miRNA expression patterns have been associated with the ability of certain individuals to spontaneously control viral replication without therapy, highlighting their regulatory influence on host antiviral responses [50]. Moreover, miRNA dysregulation has been linked to immune cell senescence and chronic inflammation, both contributing to HIV-1 disease progression, with expression profiles varying depending on the timing and efficacy of antiretroviral therapy [51]. While some miRNAs like miR-28, miR-125b, miR-150, and miR-155 restrict HIV-1 replication in macrophages, the virus has evolved mechanisms to manipulate host miRNA pathways, such as viral protein *Vpu* inducing miR-25 and miR-93 to suppress host restriction factors [52], [53].

The microRNA-mediated regulatory effects in macrophages are particularly relevant given that HIV-1, the retrovirus responsible for a global pandemic, has evolved sophisticated mechanisms to circumvent host immunity [54], [55]. HIV-1 targets CD4 cells and has adapted to use macrophages as viral reservoirs. While most T cells are depleted upon HIV-1 infection, macrophages are resistant to virus-mediated killing and survive to form reservoirs that contribute to viral recrudescence [56], [57], [58]. Recent evidence supports the capability of macrophages in transmitting HIV to other macrophages or T cells *via* cell–cell contact, thereby efficiently spreading the virus [59], [60]. The formation of latent cellular reservoirs remains a major hurdle in preventing HIV-1 eradication, and the complex mechanisms by which macrophages contribute to HIV-1 persistence are still largely unknown [61], [62]. While it is partly recognized that HIV-1 regulates numerous pathways in macrophages upon infection, including those involved in immune response, apoptosis, and cellular metabolism, the mechanisms still require further understanding [63], [64]. Given the nature of miRNAs, it is plausible that they represent a crucial ‘missing link’ in HIV-1-macrophage biology, potentially explaining some of the observed pathway dysregulations. But, thus far, the extent and implications of miRNA dysregulation in HIV-1-infected macrophages remain poorly understood [65], [66], [67]. Macrophages, representing integral cells of the immune system taking part in both innate and adaptive immune responses, demand more understanding of miRNA expression profiles upon HIV-1 infection.

In the current study, we explored the dynamics of miRNAs following HIV-1 infection in PMA-differentiated THP-1 cells, a well-established *in vitro* model for macrophages [68]. Subsequently, *in silico* database mining and network analysis predicted hub target proteins and the altered pathways in HIV-1 infected macrophages with the goal of identifying potential therapeutic targets and better understanding the role of miRNAs in HIV-1 pathogenesis. With the recent approval of RNAi-based therapeutics by the FDA, understanding miRNA significance might open new avenues for HIV-1 therapy. The targeted modulation of specific miRNAs represents a promising frontier in developing novel strategies to control HIV replication and address viral reservoir persistence.

## 2 Materials and methods

### 2.1 Cell culture and differentiation

The human monocyte/macrophage type cell line THP-1 cells (ATCC: TIB-202™, ATCC, Manassas, VA, USA) were cultured in RPMI 1640 medium supplemented with 10 % fetal bovine serum (FBS) (Gibco, USA) and 1 % penicillin-streptomycin (Sigma-Aldrich, St. Louis, MO, USA), 20 mM HEPES (Gibco, USA), 1 mM Sodium pyruvate (Gibco, USA). Cells were maintained in a humidified incubator at 37°C with 5 % CO_2_. The cells were passaged when they reached approximately 80 % confluency.

For differentiation into macrophages, THP-1 cells were seeded in tissue culture plates at a density of 1 × 10^6^ cells per well. Cells were then treated with 20 nM phorbol 12-myristate 13-acetate (PMA) for 24 hours. Following treatment, the PMA-containing medium was removed, and cells were washed twice with phosphate-buffered saline (PBS). Fresh RPMI 1640 medium supplemented with 10 % FBS was then added, and the cells were allowed to rest for an additional 24 hours before further experiments.

### 2.2 Viral strain and stock expansion

The macrophage-tropic Indian primary isolate HIV-1_VB028_ (R5, Subtype C), deposited in the viral bank of the Division of Virology, ICMR-National Institute of Translational Virology and AIDS Research, Pune, was employed for the study. The virus was propagated using the phytohemagglutinin-P (PHA-P) activated Peripheral blood mononuclear cells (PBMCs). PBMCs were separated from donor blood samples through density gradient centrifugation utilizing Histopaque (Sigma-Aldrich, USA). Following separation, the PBMCs were treated with 5 μg/mL PHA-P (Sigma-Aldrich, USA) in RPMI 1640 culture medium (Gibco, USA) for its activation. The culture medium contained 10 % fetal bovine serum (FBS), antibiotics (50 U/mL penicillin and 50 mg/mL streptomycin), and was supplemented with 5 U/mL interleukin-2 (IL-2). The HIV-1 p24 antigen detection assay (Abcam, Cambridge, UK) was employed to quantify the infectivity of the viral stocks prepared using activated PBMCs. The TZM-bl cell line (HeLa-derived recombinant cell with HIV-1 LTR-driven luciferase reporter) was used for viral titration, and the Spearman-Karber method was utilized to calculate the 50 % tissue culture infective dose (TCID_50_), as previously described [69], [70], [71].

### 2.3 HIV-1 infection of THP-1 macrophage

THP-1 monocytes were differentiated into macrophages, as described earlier. THP-1 macrophages 1 × 10^6^ well were infected with HIV-1_VB028_ at a TCID_50_ of 400. The plates were maintained at 37°C, 5 % CO_2_ for 48 hours. Mock-infected controls were treated identically but without virus. Assay duplicate was used to confirm the viral infection using primers against HIV-1 Gag gene in qRT-PCR.

### 2.4 miRNA microarray

THP-1 macrophages were infected with HIV-1 for 48 h, with mock-infected cells serving as controls. Mock infection was performed by exposing cells to RPMI 1640 medium containing 10 % FBS. The dysregulation of cellular miRNAs involved in HIV-1-induced inflammation was investigated using custom-made microarray PCR plates specific for human inflammatory pathways (YAHS-205ZD:339325, Qiagen, Hilden, Germany).

Total miRNA was extracted using the *mir*Vana™ miRNA Isolation Kit as per the manufacturer’s protocol (Invitrogen, Waltham, MA, USA: AM1560). cDNA synthesis was performed using the miRCURY LNA RT Kit (Qiagen, Hilden, Germany) with 10 ng RNA as the template. The synthesized cDNA served as a template for SYBR green-based quantitative PCR (qPCR) using the miScript LNA SYBR Green PCR kit (Qiagen, Hilden, Germany). qPCR reactions were conducted under the following conditions: initial denaturation at 95°C for 2 min, followed by 40 cycles of 95°C for 10 s and 56°C for 60 s. All experiments were performed with three independent biological replicates, with RNA isolated separately from each replicate of both HIV-1 infected and mock-infected conditions. Data analysis was performed using the GeneGlobe data analysis tool from Qiagen (https://geneglobe.qiagen.com/in accessed on 17 December 2023). For differential expression analysis, miRNAs with a fold change threshold of 2.0 (upregulation) or −2.0 (downregulation) and a p-value less than 0.05 were considered statistically significant and selected for further analysis.

### 2.5 miRNA target identification

For miRNA target identification, we utilized miRTarBase (https://mirtarbase.cuhk.edu.cn: version 9.0), an experimentally validated microRNA-target interactions database. The top 5 upregulated and top 5 downregulated miRNAs identified from our microarray analysis were used as query inputs. Target genes for these miRNAs were retrieved from the database and downloaded for further analysis.

### 2.6 Protein-protein interaction

To elucidate the potential functional impact caused by affecting the identified miRNA targets, protein-protein interaction (PPI) analysis was performed. The miRNA target data from miRTarBase was cleaned and organized, and all identified target genes were compiled into a master list. Any gene that is the target of either up or down miRNA or both upregulated and downregulated miRNAs are also screened and separated. Protein-protein interaction (PPI) data were retrieved from the BIOGRID database (https://thebiogrid.org/: version 4.4.229). From the data set, unique human interactions were filtered after removing any redundant interactions detected from multiple sources. The miRNA targets were queried against the BIOGRID database to screen the interaction of miRNA targets with different human interactors. Subsequently, the protein-protein interactions with miRNA targets along with their first-degree interactors, those directly affected by these targets, were screened and used for the preparation of further interaction network.

### 2.7 Cytoscape network analysis

The interaction of miRNA targets was prepared using Cytoscape (https://cytoscape.org/: version 3.10.1). The network of upregulated, downregulated, or both miRNA targets, along with their first interactors, were extracted from combined miRNA network and analysed separately. Network was visualized through Cytoscape, and network analysis was done through an inbuilt network analyser tool. For each node, a set of network parameters was calculated to characterize its importance within the network. These parameters included degree, betweenness centrality, closeness centrality, clustering coefficient, average shortest path length, eccentricity, neighbourhood connectivity, number of directed and undirected edges, radiality, stress, and topological coefficient. The key regulatory nodes and structural features within the miRNA-mediated interaction network were identified.

### 2.8 Gene ontology and pathway enrichment analysis

In order to predict the role of these miRNA targets in modulating normal functions, the Cytoscape app GOlorize (https://apps.cytoscape.org/apps/golorize) was used in conjunction with BiNGO (https://apps.cytoscape.org/apps/bingo) to predict Gene Ontology (GO) for molecular functions. This method uses a graph layout algorithm and finds overrepresented GO categories in a network while using it in conjunction with BiNGO [72]. The network of miRNA target genes and their first-degree interactors was imported into Cytoscape, and BiNGO was used to analyze overrepresented GO molecular function terms using a hypergeometric statistical test with Benjamini & Hochberg FDR correction using Homo sapiens. A significance level of 0.05 was used to select overrepresented terms after correction, and overrepresented terms were identified and represented. The resulting network was visualized using Cytoscape version 3.10.1. Further, Pathway enrichment analysis was conducted using the Database for Annotation, Visualization, and Integrated Discovery (DAVID, v2024q2) to investigate the biological significance of differentially expressed genes identified in this study. Gene identifiers were submitted to DAVID, and enrichment was performed using three databases: KEGG, Reactome, and WikiPathways. Significantly enriched pathways were identified with the p-value.

## 3 Results

Building on our understanding of the complex interplay between HIV-1 and host immune responses and shedding light on the molecular mechanisms underlying HIV-1-induced miRNA dysregulation, we conducted a series of experiments to elucidate the effects of HIV-1 infection on miRNA expression in macrophages.

### 3.1 HIV-1 infection of macrophages induces differential expression of inflammatory response-associated miRNAs

To explore the impact of HIV-1 infection on miRNA expression in macrophages, we employed a custom panel focusing on miRNAs involved in human inflammatory responses. This targeted approach allowed us to hone in on a specific subset of miRNAs that are likely to play crucial roles in the host immune response. The miRNA assay was normalized using a factor calculated based on multiple reference miRNAs included in the panel, ensuring accuracy in the fold-change calculations, which were performed using the GeneGlobe data analysis tool. The differential expression of miRNAs (represented as fold changes) is summarized in Table 1.

**Table 1:** The differential expression of miRNA in Macrophages upon HIV-1 Infection.

Sl. No	Position	miRNA ID	Fold Regulation	p-Value
1	A01	hsa-let−7a−5p	−1.59	0.089901
2	A02	hsa-let−7b−5p	−1.34	0.003203
3	A03	hsa-let−7c−5p	−1.56	0.00178
4	A04	hsa-let−7d−5p	−1.48	0.002133
5	A05	hsa-let−7e−5p	−1.63	0.001579
6	A06	hsa-let-7f−5p	−1.56	0.001805
7	**A07**	**hsa-let-7g−5p**	**−3.56**	**0.001253**
8	A08	hsa-let−7i−5p	−1.05	0.030642
9	A09	hsa-miR−101–3p	−1.58	0.001702
10	A10	hsa-miR−106b−5p	−1.29	0.004025
11	A11	hsa-miR−125a−5p	−1.25	0.004698
12	**A12**	**hsa-miR−125b−5p**	**8.30**	**0.000019**
13	B01	hsa-miR−128–3p	1.43	0.00229
14	B02	hsa-miR−130a−3p	1.70	0.003478
15	B03	hsa-miR−130b−3p	1.88	0.000507
16	B04	hsa-miR−1324	1.80	0.000611
17	**B05**	**hsa-miR−144–3p**	**6.82**	**0.000023**
18	**B06**	**hsa-miR−145–5p**	**3.19**	**0.000865**
19	**B07**	**hsa-miR−15a−5p**	**−2.40**	**0.036067**
20	**B08**	**hsa-miR−15b−5p**	**−2.72**	**0.001367**
21	B09	hsa-miR−16–5p	−1.50	0.002051
22	B10	hsa-miR−17–5p	1.04	0.383563
23	B11	hsa-miR−181a−5p	1.72	0.000766
24	**B12**	**hsa-miR−181b−5p**	**2.27**	**0.007459**
25	C01	hsa-miR−181c−5p	1.80	0.000611
26	**C02**	**hsa-miR−181d−5p**	**−2.62**	**0.045591**
27	C03	hsa-miR−186–5p	1.52	0.001512
28	**C04**	**hsa-miR−195–5p**	**−2.18**	**0.000851**
29	C05	hsa-miR−19a−3p	−1.29	0.003897
30	C06	hsa-miR−19b−3p	−1.25	0.004655
31	**C07**	**hsa-miR−202–3p**	**5.85**	**0.041074**
32	C08	hsa-miR−20a−5p	1.05	0.479648
33	C09	hsa-miR−20b−5p	1.28	0.006225
34	C10	hsa-miR−21–5p	−1.65	0.001527
35	**C11**	**hsa-miR−211–5p**	**−4.72**	**0.009209**
36	C12	hsa-miR−23a−3p	−1.52	0.001936
37	D01	hsa-miR−23b−3p	−1.57	0.00176
38	D02	hsa-miR−29a−3p	1.40	0.002706
39	**D03**	**hsa-miR−29b−3p**	**−2.44**	**0.001336**
40	D04	hsa-miR−29c−3p	1.01	0.103692
41	**D05**	**hsa-miR−300**	**9.69**	**0.003237**
42	D06	hsa-miR−301a−3p	−1.09	0.015615
43	D07	hsa-miR−301b−3p	1.16	0.025928
44	D08	hsa-miR−302a−3p	1.80	0.000611
45	D09	hsa-miR−302b−3p	1.80	0.000611
46	D10	hsa-miR−302c−3p	1.80	0.000611
47	D11	hsa-miR−30a−5p	1.33	0.004133
48	D12	hsa-miR−30b−5p	−1.61	0.001634
49	**E01**	**hsa-miR−30c−5p**	**−2.09**	**0.000910**
50	E02	hsa-miR−30d−5p	1.38	0.003115
51	E03	hsa-miR−30e−5p	−1.15	0.008853
52	E04	hsa-miR−340–5p	−1.64	0.001540
53	E05	hsa-miR−34a−5p	1.36	0.003538
54	**E06**	**hsa-miR−34c−5p**	**7.49**	**0.000021**
55	E07	hsa-miR−372–3p	1.80	0.000611
56	E08	hsa-miR−373–3p	1.80	0.000611
57	**E09**	**hsa-miR−374a−5p**	**−2.59**	**0.008858**
58	E10	hsa-miR−381–3p	1.80	0.000611
59	**E11**	**hsa-miR−410–3p**	**−4.63**	**0.048088**
60	**E12**	**hsa-miR−424–5p**	**−2.88**	**0.006601**
61	F01	hsa-miR−449a	1.80	0.000611
62	F02	hsa-miR−449b−5p	1.80	0.000611
63	**F03**	**hsa-miR−454–3p**	**−3.79**	**0.006141**
64	F04	hsa-miR−497–5p	−1.01	0.061146
65	F05	hsa-miR−511–5p	1.80	0.000611
66	F06	hsa-miR−513b−5p	1.80	0.000611
67	F07	hsa-miR−519c−3p	1.80	0.000611
68	F08	hsa-miR−519d−3p	1.80	0.000611
69	**F09**	**hsa-miR−520d−3p**	**11.11**	**0.032702**
70	F10	hsa-miR−520e−3p	1.80	0.000611
71	F11	hsa-miR−524–5p	1.80	0.000611
72	**F12**	**hsa-miR−543**	**−3.00**	**0.045975**
73	**G01**	**hsa-miR−545–3p**	**−7.43**	**0.036831**
74	G02	hsa-miR−548c−3p	1.80	0.000611
75	G03	hsa-miR−548d−3p	−1.30	0.003851
76	G04	hsa-miR−548e−3p	1.80	0.000611
77	G05	hsa-miR−590–5p	−1.22	0.005523
78	G06	hsa-miR−607	1.80	0.000611
79	G07	hsa-miR−655–3p	1.80	0.000611
80	G08	hsa-miR−656–3p	1.80	0.000611
81	G09	hsa-miR−875–3p	1.80	0.000611
82	**G10**	**hsa-miR−9–5p**	**−3.25**	**0.046856**
83	G11	hsa-miR−93–5p	1.42	0.002448
84	G12	hsa-miR−98–5p	−1.33	0.003387

Bold denotes downregulation and upregulation with statistical significance.

**Fig. 1:**
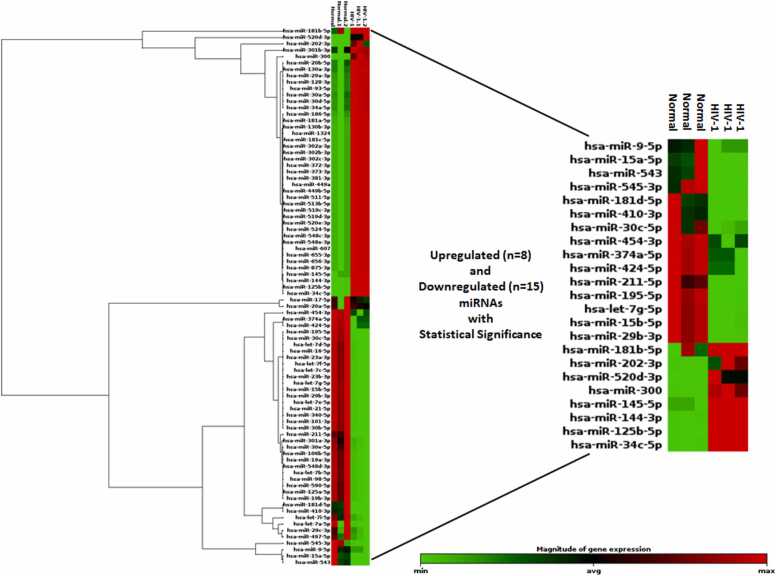
**Heat map of miRNA expression in HIV-1 infected*****vs*****uninfected macrophages.** Expression levels of 84 miRNAs associated with the human inflammatory response pathway were analyzed using a focused miRNA microarray assay. Red indicates upregulation, green indicates downregulation, with color intensity corresponding to magnitude of change. The data derived from three independent experimental replicates to maintain precision of the results. 23 miRNAs showed differential expression between control and HIV-1 infected samples with statistical significance. Differential expression of 8 miRNAs were found to be elevated and 15 were suppressed in HIV-1 infected cells. The analysis used a cut-off of at least 2-fold changes.

Our analysis revealed that 23 out of 84 miRNAs were dysregulated in macrophages following HIV-1 infection. Among these, 8 miRNAs were upregulated, and 15 were downregulated, with a cut-off of at least a 2-fold change applied to define significant dysregulation. Visualization of the differential expression data was conducted through a heat map. The heat map distinctly categorized the miRNAs into two groups: those with reduced expression (depicted in green) and those with elevated expression (shown in red) in response to HIV-1 infection. The intensity of these colours was directly proportional to the magnitude of the expression changes (Fig. 1).

**Fig. 2:**
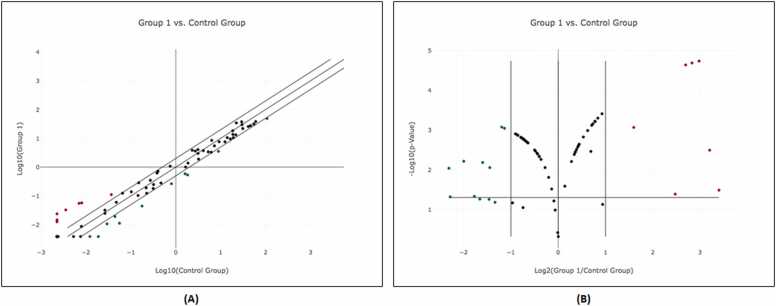
**Differential miRNA expression in HIV-1 infected macrophages compared to uninfected controls**. (A) Scatter plot analysis of miRNA expression. The plot compares log10 transformed expression values between HIV-1 infected and uninfected control macrophages. Each point represents an individual miRNA. The central diagonal line indicates no change in expression, while the outer lines represent 2-fold up- or down-regulation thresholds. Green points indicate downregulated miRNAs, black points show unchanged expression, and red points represent upregulated miRNAs in HIV-1 infected macrophages. (B) Volcano plot depicting statistical significance and magnitude of miRNA expression changes. The x-axis shows Log2(Group 1/Control Group) fold change, and the y-axis represents -Log10(p-value). Vertical lines denote 2-fold up- and down-regulation thresholds, while the horizontal line indicates the p-value significance threshold. Red points represent statistically significant upregulated miRNAs, green points indicate statistically significant downregulated miRNAs, and black points represent miRNAs with non-significant changes or less than 2-fold change.

While the heat map clearly illustrates the polarized expression patterns of miRNAs between the HIV-1-infected and mock-infected macrophages, with distinct differences in expression levels, the scatter plot provides a detailed view of the magnitude of miRNA expression differences between the control group and the HIV-1-infected group (Group 1), plotted on a logarithmic scale (Fig. 2A). Each point on the scatter plot represents the expression of a specific miRNA, comparing the control (X-axis) to the HIV-1-infected group (Y-axis). Additionally, the volcano plot (Fig. 2B) highlights the statistical significance of these expression changes by plotting the p-value against the magnitude of fold change. This representation underscores the statistical reliability of the data derived from three independent replicates and enhances the reproducibility and precision of the results. The use of these visualizations ensures a robust selection of miRNAs for further study, emphasizing both the degree of dysregulation and the statistical significance of the observed changes.

### 3.2 miRTarBase analysis mapped 10 most significantly dysregulated miRNAs to over 2000 experimentally validated unique gene targets

To assess the potential functional consequences of the differentially expressed miRNAs in HIV-1-infected macrophages, we performed target identification analysis using miRTarBase (version 9.0), a comprehensive database of experimentally validated miRNA-target interactions. We focused on the 5 most upregulated and 5 most downregulated miRNAs out of the 23 dysregulated miRNAs identified in this study. For the upregulated miRNAs, we identified 1358 target entries, corresponding to 1258 unique genes. The miRNA with the highest number of target entries was hsa-miR-125b-5p, with 463 targets, followed by hsa-miR-520d-3p (435 targets), hsa-miR-144–3p (204 targets), hsa-miR-34c-5p (144 targets), and hsa-miR-300 (112 targets). In the case of the downregulated miRNAs, we identified 1212 target entries corresponding to 1122 unique targets. The miRNA with the highest number of target entries was hsa-miR-454–3p, with 373 targets, followed by hsa-miR-211–5p (330 targets), hsa-miR-410–3p (245 targets), hsa-miR-545–3p (153 targets), and hsa-miR-543 (111 targets). Combining the targets of both upregulated and downregulated miRNAs, we identified a total of 2570 target entries, corresponding to 2159 unique targets. The presence of 221 duplicate entries across the dataset suggests that several genes are targeted by multiple dysregulated miRNAs. The complete list of these targets is available as Supplementary Data (Combined Analysis: Sheet 1–4).

### 3.3 BIOGRID protein-protein interaction analysis reveals extensive network influenced by HIV-1-induced miRNA dysregulation

To further elucidate the impact of HIV-1-induced miRNA dysregulation on cellular processes, we conducted a protein-protein interaction (PPI) analysis using the BIOGRID database. Out of 893,638 unique human interactions, 283,053 (31.7 %) involved interactions with the targets of the studied miRNAs (n = 10). Notably, 28,626 of these interactions were first-degree interactors of targets that were also directly regulated by the 10 most dysregulated miRNAs identified in our study (Supplementary Data - Combined Analysis: Sheet 5–8). These findings suggest that HIV-1-induced miRNA dysregulation may exert wide-ranging effects on cellular functions, potentially influencing a substantial portion of the human interactome.

### 3.4 Network analysis identified key regulatory nodes in HIV-1-induced miRNA target network

We performed a detailed network analysis using Cytoscape to visualize and interpret the protein-protein interactions among the miRNA targets and their first interactors. This analysis revealed a complex and highly interconnected network of interactions. The most highly connected node in this network was *ZRANB1*, a target of downregulated miRNAs, with a degree of 4172. This was followed by *MYC* (degree: 3189) and *KRAS* (degree: 2919), both of which were targets of upregulated miRNAs. Other key nodes identified included *CUL3* (degree: 2798), *TP53* (degree: 2721), *APP* (degree: 2638), and *ESR1* (degree: 2479).

Interestingly, several nodes such as *KRAS*, *TP53*, *ESR1*, *CUL3*, *PARP1*, and *GSK3B* were found to be targets of both upregulated and downregulated miRNAs. This dual targeting suggests a complex regulatory landscape where these genes are subject to multifaceted control by miRNAs influenced by HIV-1 infection. The regulatory significance of these nodes was further explored by calculating disease node scores, which considered network parameters such as degree, betweenness centrality (BC), clustering coefficient (CC), and average shortest path length (ASPL) [73], normalized using specific formulas. Therefore, disease node scores were calculated following the normalization of network parameters value using the following formula in the combined target network.

$$\mathrm{Disease node score}=\;\frac{{\mathrm{Degree}}_{\mathrm{norm}}+\;{\mathrm{BC}}_{\mathrm{norm}}}{{\mathrm{CC}}_{\mathrm{norm}}+\;{\mathrm{ASPL}}_{\mathrm{norm}}}\;$$

Where the normalization was represented as

$${\mathrm{Degree}}_{\mathrm{norm}}=\;\frac{{\mathrm{Degree}}_{\mathrm{node}}-\;{\mathrm{Degree}}_{\min}}{{\mathrm{Degree}}_{\max}-\;{\mathrm{Degree}}_{\min}}$$

$${\mathrm{BC}}_{\mathrm{norm}}=\;\frac{{\mathrm{BC}}_{\mathrm{node}}-\;{\mathrm{BC}}_{\min}}{{\mathrm{BC}}_{\max}-\;{\mathrm{BC}}_{\min}}$$

$${\mathrm{CC}}_{\mathrm{norm}}=\;\frac{{\mathrm{CC}}_{\mathrm{node}}-\;{\mathrm{CC}}_{\min}}{{\mathrm{CC}}_{\max}-\;{\mathrm{CC}}_{\min}}$$

$${\mathrm{ASPL}}_{\mathrm{norm}}=\;\frac{{\mathrm{ASPL}}_{\mathrm{node}}-\;{\mathrm{ASPL}}_{\min}}{{\mathrm{ASPL}}_{\max}-\;{\mathrm{ASPL}}_{\min}}$$

**Fig. 3:**
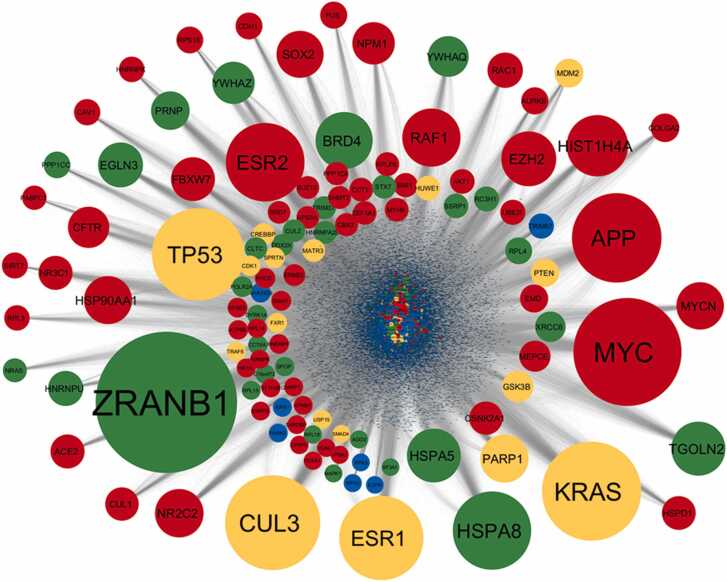
**Interaction network of miRNA targets and their first interactors as per interaction data obtained from BIOGRID 4.4.229.** This network visualization represents upregulated miRNA targets with red color, downregulated miRNA targets with green color, both up and down miRNA targets with orange color, and other human targets with blue color nodes. The size of the node is arranged as per their relative degree value in the interaction network.

The interaction network of miRNA targets was constructed to visualize the broader impact of HIV-1-induced miRNA dysregulation on cellular processes. The network analysis identified several highly interconnected nodes, which are indicative of critical regulatory hubs within the network. These hubs, or disease nodes, are particularly significant as they may represent key points of vulnerability or control within the cell's response to HIV-1 infection. To further refine our analysis, the disease nodes were categorized based on a calculated score. These top-ranked disease nodes (Table 2) provide a clear view of the most influential targets within the network (Fig. 3), representing potential therapeutic targets, as their dysregulation could have widespread effects on cellular function.

**Table 2:** Top 20 genes in the combined network ranked according to the disease score.

**Sl. No.**	**Gene**	**Disease node Score**	**Targeting miRNA Expression**
1	ZRANB1	4.700070283	Downregulated
2	KRAS	3.35972315	Both
3	APP	3.199559451	Upregulated
4	CUL3	2.77976849	Both
5	MYC	2.749710376	Upregulated
6	TP53	2.552078059	Both
7	HSPA8	1.989861198	Downregulated
8	ESR2	1.932012165	Upregulated
9	ESR1	1.88556316	Both
10	TGOLN2	1.439907778	Downregulated
11	HSPA5	1.426698995	Downregulated
12	RAF1	1.183360563	Upregulated
13	HIST1H4A	1.177426293	Upregulated
14	HSP90AA1	1.061589466	Upregulated
15	EZH2	1.059684953	Upregulated
16	CFTR	1.054236071	Upregulated
17	BRD4	1.025858166	Downregulated
18	PRNP	0.910649715	Downregulated
19	SOX2	0.892597337	Upregulated
20	YWHAZ	0.872974495	Downregulated

**Fig. 4:**
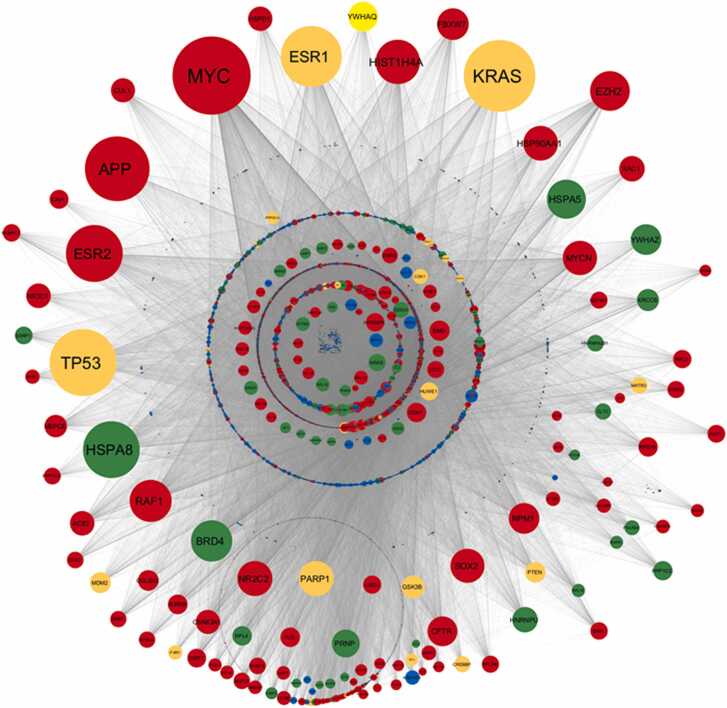
**Interaction network of upregulated miRNA targets and their first interactors as per interaction data obtained from BIOGRID 4.4.229.** This network visualization represents upregulated miRNA targets with red color, downregulated miRNA targets with green color, both up and down miRNA targets with orange color, and other human targets with blue color nodes. The size of the node is arranged as per their relative degree value in the interaction network. Upregulated miRNA targets also display interaction with other miRNAs targets and therefore present different color nodes in interaction network.

**Fig. 5:**
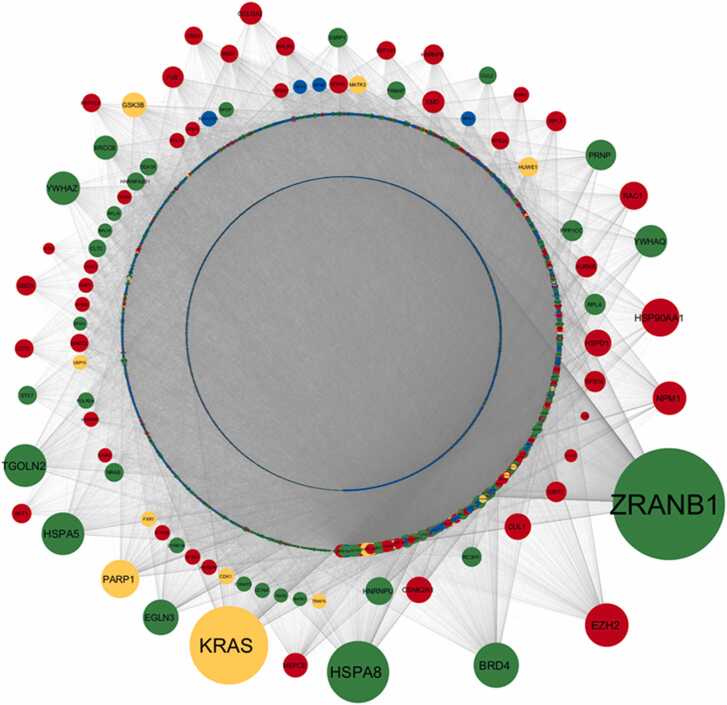
**Interaction network of downregulated miRNA targets and their first interactors as per interaction data obtained from BIOGRID 4.4.229.** This network visualization represents upregulated miRNA targets with red color, downregulated miRNA targets with green color, both up and down miRNA targets with orange color, and other human targets with blue color nodes. The size of the node is arranged as per their relative degree value in the interaction network. Downregulated miRNA targets also display interaction with other miRNAs targets and therefore present different color nodes in interaction network.

The next phase of our analysis focused on the interaction network specific to the upregulated miRNA target genes. This network, formed by these targets and their first interactors, reveals the immediate downstream effects of miRNA upregulation in HIV-1-infected macrophages. This network sheds light on the potential pathways and processes that are most affected by the upregulated miRNAs. To prioritize the most significant nodes within this network, we observed the degree of the nodes, which highlights the key regulatory elements that might be driving the observed changes in cellular function (Fig. 4, Table 3). Similarly, the interaction network for downregulated miRNA target genes was constructed to understand the consequences of reduced miRNA expression in the context of HIV-1 infection. This network illustrates how the downregulated miRNAs influence their immediate interactors and potentially disrupt normal cellular functions. The identification of key nodes within this downregulated miRNA target network was achieved through the same system used for the upregulated targets, highlighting the most critical targets affected by the downregulated miRNAs. These targets are particularly important as they may represent areas where loss of regulation contributes to HIV-1 pathology (Fig. 5, Table 4).

**Table 3:** Most 20 genes that are the targets of up regulated miRNA and its First interactor.

**Sl. No.**	**Gene**	**Degree**	**Targeting miRNA Expression**
1	MYC	3189	Upregulated
2	KRAS	2919	Both
3	TP53	2721	Both
4	APP	2638	Upregulated
5	ESR1	2479	Both
6	ESR2	2316	Upregulated
7	HSPA8	2305	Downregulated
8	HIST1H4A	1805	Upregulated
9	RAF1	1710	Upregulated
10	BRD4	1675	Downregulated
11	EZH2	1614	Upregulated
12	HSPA5	1579	Downregulated
13	NR2C2	1423	Upregulated
14	PARP1	1412	Both
15	HSP90AA1	1409	Upregulated
16	MYCN	1392	Upregulated
17	SOX2	1381	Upregulated
18	FBXW7	1286	Upregulated
19	NPM1	1242	Upregulated
20	YWHAZ	1236	Downregulated

**Table 4:** Most 20 genes that are the targets of down regulated miRNA and its First interactor.

**Sl. No.**	**Gene**	**Degree**	**Targeting miRNA Expression**
1	ZRANB1	4172	Downregulated
2	KRAS	2919	Both
3	HSPA8	2305	Downregulated
4	BRD4	1675	Downregulated
5	EZH2	1614	Upregulated
6	TGOLN2	1584	Downregulated
7	HSPA5	1579	Downregulated
8	PARP1	1412	Both
9	HSP90AA1	1409	Upregulated
10	EGLN3	1315	Downregulated
11	NPM1	1242	Upregulated
12	YWHAZ	1236	Downregulated
13	YWHAQ	1190	Downregulated
14	PRNP	1125	Downregulated
15	RAC1	1034	Upregulated
16	HNRNPU	1023	Downregulated
17	HSPD1	987	Upregulated
18	CUL1	976	Upregulated
19	CSNK2A1	974	Upregulated
20	GSK3B	912	Both

**Fig. 6:**
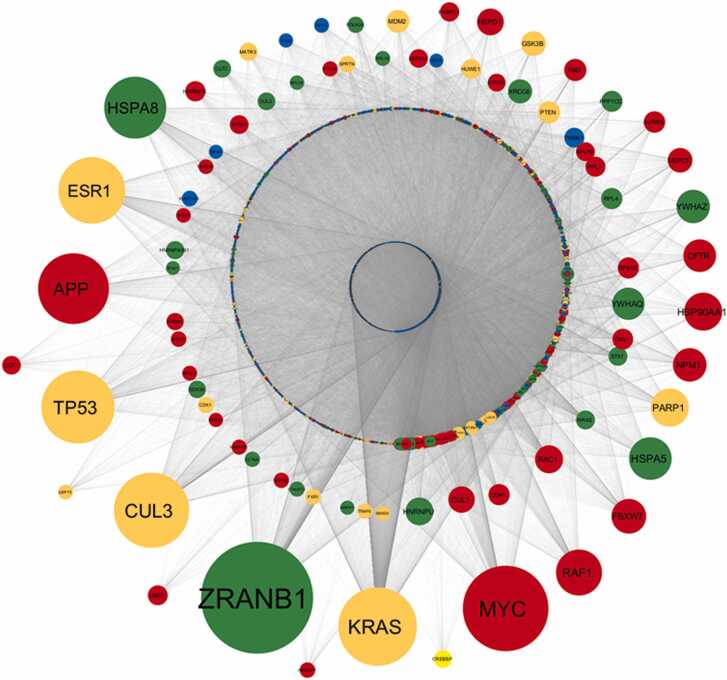
**Interaction network of targets that are concurrently regulated by both upregulated and downregulated miRNAs and their first interactors as per interaction data obtained from BIOGRID 4.4.229.** This network visualization represents upregulated miRNA targets with red color, downregulated miRNA targets with green color, both up and down miRNA targets with orange color, and other human targets with blue color nodes. The size of the node is arranged as per their relative degree value in the interaction network. Targets of both up and downregulated miRNA also display interaction with other miRNAs targets and therefore present different color nodes in interaction network.

Finally, to capture the full scope of HIV-1-induced miRNA dysregulation, we analyzed the network of miRNA targets that are concurrently regulated by both upregulated and downregulated miRNAs. This network showcases the complex interplay between various miRNA targets and their interactors, providing a holistic view of the cellular landscape under HIV-1 infection. The network highlights potential areas of crosstalk and interaction between different miRNA pathways. The presence of common targets across both upregulated and downregulated networks underscores the complexity of HIV-1's impact on macrophage function and suggests potential points of intersection where therapeutic interventions might be most effective (Fig. 6, Table 5).

**Table 5:** Most 20 genes that are the targets of both up and down regulated miRNA and its First interactor.

**Sl. No.**	**Gene**	**Degree**	**Targeting miRNA Expression**
1	ZRANB1	4172	Downregulated
2	MYC	3189	Upregulated
3	KRAS	2919	Both
4	CUL3	2798	Both
5	TP53	2721	Both
6	APP	2638	Upregulated
7	ESR1	2479	Both
8	HSPA8	2305	Downregulated
9	RAF1	1710	Upregulated
10	HSPA5	1579	Downregulated
11	PARP1	1412	Both
12	HSP90AA1	1409	Upregulated
13	FBXW7	1286	Upregulated
14	NPM1	1242	Upregulated
15	YWHAZ	1236	Downregulated
16	CFTR	1210	Upregulated
17	YWHAQ	1190	Downregulated
18	RAC1	1034	Upregulated
19	HNRNPU	1023	Downregulated
20	HSPD1	987	Upregulated

### 3.5 Gene ontology analysis reveals enrichment of significant molecular functions dysregulated by HIV-1-induced miRNA changes

**Fig. 7:**
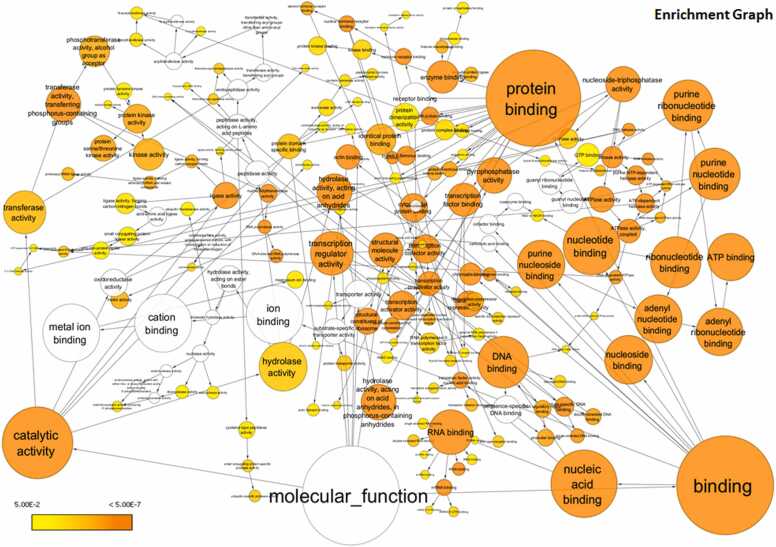
**Upregulated miRNA target and their first interactors analysis for Gene ontology:** Molecular function using BINGO and visualized through Cytoscape 3.10.1. The colour of the nodes represents the significance of the over-representation as indicated in the scale bar, where colour towards yellow to orange indicate increasing significance.

**Fig. 8:**
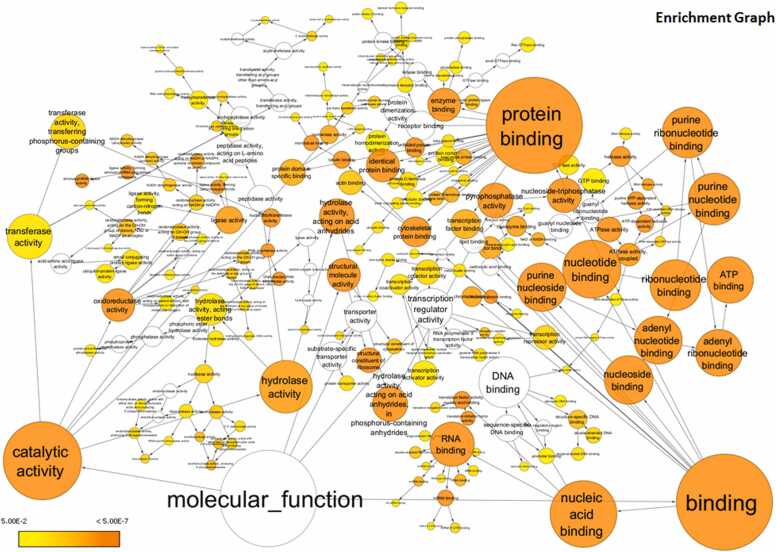
**Downregulated miRNA target and their first interactors analysis for Gene ontology:** Molecular function using BINGO and visualized through Cytoscape 3.10.1. The colour of the nodes represents the significance of the over-representation as indicated in the scale bar, where colour towards yellow to orange indicate increasing significance.

To gain further insights into the molecular functions impacted by the dysregulated miRNAs, we performed the Gene Ontology (GO) analysis. The analysis revealed significant enrichment of several critical molecular functions associated with the targets of both upregulated miRNAs (Fig. 7) and downregulated miRNAs (Fig. 8).

**Fig. 9:**
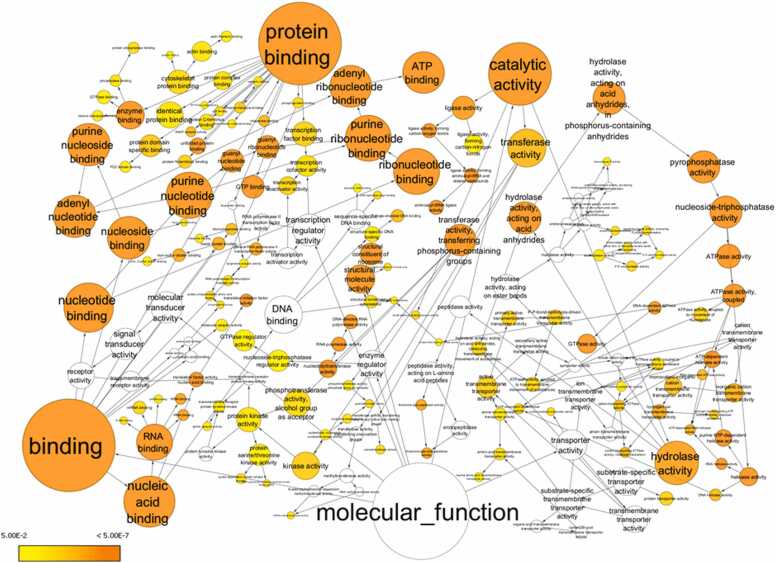
**Both up and downregulated miRNA target and their first interactors analysis for Gene ontology:** molecular function using BINGO and visualized through Cytoscape 3.10.1. The colour of the nodes represents the significance of the over-representation as indicated in the scale bar, where colour towards yellow to orange indicate increasing significance.

Binding activities emerged as one of the most significantly affected categories. Specifically, the most enriched terms included protein binding, RNA binding, purine ribonucleotide binding, ribonucleotide binding, and structural components of ribosomes. Other highly enriched functions included nucleotide binding, purine nucleotide binding, ATP binding, adenyl ribonucleotide binding, nucleoside binding, adenyl nucleotide binding, and nucleoside-triphosphatase activity. Additional enriched functions were related to purine nucleoside binding, pyrophosphatase activity, hydrolase activity on acid anhydrides, ATPase activity, coupled ATPase activity, and RNA polymerase activity. These findings suggest that HIV-1-induced miRNA dysregulation in macrophages could have far-reaching consequences, potentially disrupting a broad range of cellular processes, including protein-protein interactions, RNA metabolism, nucleotide binding and hydrolysis, and ribosome structure and function. The enriched functions are visually represented for the upregulated miRNA targets (Fig. 7), downregulated miRNA targets (Fig. 8), and the combined set of targets for both upregulated and downregulated miRNAs along with their first interactors (Fig. 9), showcasing the top molecular functions sorted by enrichment scores.

### 3.6 Pathway enrichment analysis revealed the key pathways influenced by the most dysregulated miRNAs

**Fig. 10:**
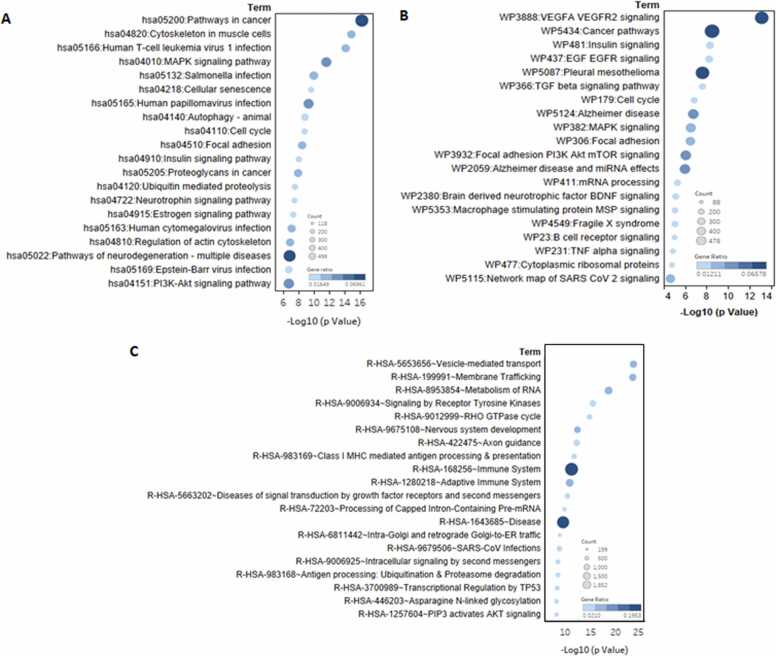
**DAVID pathway functional annotation analysis of targets against pathway databases**: **(A)** KEGG, **(B)** WikiPathways, and **(C)** Reactome Pathways. The top 20 pathways are presented based on their significance, measured as – Log10 (p-value). The size of the data points represents the gene count, indicating the minimum number of genes associated with the corresponding pathway. The color gradient represents the gene ratio, defined as the proportion of genes in the given pathway relative to the total list. Darker shades indicate higher gene ratios. Scale bars are included for size (gene count) and color (gene ratio) to provide a clear visual reference for interpretation.

The DAVID analysis revealed several enriched pathways impacted by the genes predicted to be regulated by the dysregulated miRNAs during HIV-1 infection in monocyte-derived macrophages (MDMs). Pathway enrichment analysis was conducted using KEGG, Reactome, and WikiPathways databases, revealing a total of 210, 549, and 209 enriched pathways, respectively study (Supplementary Data - Combined Analysis: Sheet 17–19). KEGG analysis revealed dysregulations in critical pathways, including Pathways in Cancer, MAPK signaling, PI3K-Akt signaling, mTOR signaling, NF-κB signaling, cellular senescence and p53 signaling, which are integral to cell survival, proliferation, immune response, and apoptosis Pathways related to autophagy and cellular senescence were also notably affected, emphasizing the potential role of miRNA dysregulation in cellular homeostasis during HIV-1 infection. The reactome analysis provided complementary findings, including significant enrichment in pathways regulating the immune system, such as cytokine signaling, antigen presentation, and apoptosis, highlighting dysregulation of immune defense mechanisms. WikiPathways analysis identified similar critical pathways, including cancer pathways, MAPK signaling, and PI3K AKT mTOR signaling. The other pathways that are affected include VEGFR2 signaling, and Alzheimer’s disease, alongside the critically important B cell receptor signaling and TNF alpha signaling. The top 20 pathways enriched in DAVID functional annotation according to –Log10 (p-Value) is represented in Fig. 10. Collectively, these findings demonstrate the extensive influence of HIV-1-associated miRNA dysregulation on fundamental biological processes, signaling pathways, and host-pathogen dynamics, providing valuable insights into the molecular framework of HIV pathogenesis.

## 4 Discussion

MicroRNAs serve as molecular orchestrators that regulate a diverse array of functions. Their dynamic expression determines the fate of cellular activities, adapting to changes in the cellular environment, such as viral infections. This study aimed to elucidate the impact of HIV-1 infection on miRNA expression in macrophages and to explore the downstream effects of miRNA dysregulation on protein-protein interactions and molecular functions within these cells. Our investigation revealed that HIV-1 infection led to the dysregulation of 23 miRNAs associated with inflammatory responses, with 15 miRNAs downregulated and 8 miRNAs upregulated. However, it should be acknowledged that the observed miRNA expression profiles could be influenced not only by direct HIV-1 infection but also by other cellular mechanisms such as immune response variability, cellular stress responses, and epigenetic modifications. Future studies integrating transcriptomic, proteomic, and epigenomic data may offer a more holistic understanding of the underlying regulatory dynamics. The miRNAs identified in this study, which have been previously implicated in various cellular processes, including immune regulation, apoptosis, and cellular metabolism [42], suggest that their dysregulation could have far-reaching consequences for macrophage function.

Target identification analysis using miRTarBase revealed that the 10 most significantly dysregulated miRNAs were mapped to over 2000 experimentally validated unique gene targets. This extensive targeting emphasizes the potential of miRNAs to modulate a broad array of cellular functions. The identification of multiple genes targeted by both upregulated and downregulated miRNAs highlights the complex regulatory nature of miRNAs, where they may exert both activating and inhibitory effects on the same pathways, depending on their level of expression.

Protein-protein interaction analysis showed that approximately 31.7 % of human protein interactions involve targets of the most dysregulated miRNAs, according to the BIOGRID database. Although no database is complete, and information about interactions is still growing, the large number of interactions with miRNA targets indicates an extensive network influenced by HIV-1-induced miRNA dysregulation. Analyzing the node based on BC, ASPL, and CC, in addition to degree, revealed several significant nodes. The presence of key cellular regulators, such as *MYC*, *TP53*, and *KRAS*, as central hubs in our analysis, suggests that HIV-1-induced miRNA dysregulation may have extensive effects on fundamental cellular processes, including cell cycle control, apoptosis, and signal transduction. This finding is consistent with previous reports that have investigated and confirmed the deregulation of these cellular processes in HIV-1 infection [74], [75], [76].

One of the genes identified with the highest degree is *ZRANB1*, which encodes the TRABID protein and is targeted by the downregulated miRNA hsa-miR-410–3p. Recent studies have highlighted this protein's role in regulating autophagy and mitosis in cancers, which could have implications for HIV-1 pathogenesis [77]. Inhibition of TRABID has been reported to activate cGAS/STING, induce type I interferon genes, and subsequently upregulate interferon-stimulatory genes (ISGs) [78]. However, as identified in our study, *ZRANB1* is targeted by a downregulated miRNA, suggesting that its expression may be elevated compared to uninfected macrophages, potentially suppressing the activation of the cGAS/STING pathway. This finding could explain the absence of cGAS-mediated responses upon HIV-1 infection, as reported in previous studies [79], [80].

Another significant hub gene identified in this study is *MYC*, a direct target of the upregulated miRNA hsa-miR-34c-5p. This interaction suggests a potential downregulation of *MYC* expression upon HIV-1 infection of macrophages. *MYC*, a member of the basic helix-loop-helix leucine zipper (bHLH-Zip) transcription factor family, is essential for cellular growth, proliferation, and differentiation [81]. Previous studies have shown that ectopic expression of c-Myc results in decreased HIV-1 expression [82]. Conversely, it can be hypothesized that the reduced expression of MYC during HIV-1 infection may attenuate the repression of HIV-1, potentially facilitating viral replication and propagation.

Some central hub genes, like *KRAS*, *TP53*, and *CUL3*, are controlled by both upregulated and downregulated miRNAs. This dual regulation may exist because these genes control many pathways, and any skewed changes from single-sided regulation could potentially disrupt multiple cellular processes. It is possible that this bidirectional control allows protein levels to remain in dynamic states, though the exact reasons for this complex regulatory mechanism are not fully understood.

Gene ontology analysis revealed that binding activities were among the most significantly affected categories, with significant enrichment of molecular functions related to binding activities, particularly those associated with protein binding, RNA binding, and nucleotide binding. These changes in binding functions may influence various stages of the viral lifecycle, including entry, reverse transcription, integration, and assembly. Altered protein binding could affect viral-host interactions crucial for infection and replication [83]. Changes in RNA binding might impact viral RNA processing and trafficking, while differences in nucleotide binding could influence reverse transcription and integration processes [84], [85]. The enrichment of ATPase activity suggests impacts on energy metabolism, which is central to many cellular activities. Altered ATPase activity could affect energy-dependent processes critical for viral replication, such as reverse transcription, nuclear import of the viral genome, and virion assembly [86]. Moreover, changes in ATPase activity might influence cellular processes like immune signaling and antigen presentation, which are vital for the macrophage's role in immune responses [87].

As a retrovirus, HIV heavily depends on the host cell's transcriptional machinery, particularly RNA polymerase activity, to replicate its genome and produce viral proteins. GO analysis also highlighted RNA polymerase activities that may alter the transcriptional regulation in HIV-1-infected macrophages, impacting both host and viral gene expression. In protein synthesis, ribosomes play a central role crucial for both host cellular functions and viral replication, and the study revealed that the structural components of the ribosome were significantly enriched, suggesting that HIV-1-induced miRNA changes might have a more direct impact on ribosomal function than previously thought. This indicates alterations in the translation machinery, potentially affecting the efficiency or specificity of protein production. Such changes might influence the balance between host and viral protein synthesis, impacting viral replication rates, the production of immune mediators, and the overall cellular response to infection. These findings align with the well-established understanding that HIV-1 exploits and modifies the host cell's translation mechanisms to facilitate its replication and survival [88]. Another critical molecular function enriched by GO is hydrolase activity. Hydrolases catalyze the breakdown of various biological molecules and play crucial roles in numerous cellular functions, including protein degradation, lipid metabolism, and signal transduction [89]. Their enrichment could be related to changes in these processes, potentially affecting viral protein processing, cellular defense mechanisms, or the breakdown of viral components. Collectively, these alterations could either facilitate or hinder HIV-1 infection and replication in macrophages, potentially affecting viral latency and the cell's immune responses. However, further experimental investigation is required to determine whether these enriched binding activities ultimately promote or inhibit HIV-1 infection in macrophages.

The DAVID functional enrichment analysis revealed the potential dysregulation of multiple critical pathways mediated by miRNA alterations during HIV-1 infection in macrophages. The analysis highlighted that the HIV-1 pathogenesis is a multifaceted process driven by huge interactions between the virus and host cellular pathways, with dysregulated miRNAs emerging as critical regulators. Among the enriched pathways, "Pathways in Cancer" was identified as a significantly enriched term. Cancer pathways encompass numerous signaling pathway networks involved in cell cycle regulation, apoptosis, and immune evasion – the networks frequently co-opted by HIV-1 to sustain its replication and persistence. This overlap suggests that HIV-1 infection may potentially elevate the risk of malignancies in long-term infections through the involvement of miRNAs, aligning with established evidence linking chronic HIV-1 infection to a higher susceptibility to cancer [90], [91], [92], [93]. Other significantly enriched pathways included the MAPK signaling pathway, which governs cell survival, inflammation, stress response and apoptosis. MAPK is also involved in HIV-1 progression, where viral proteins, such as Tat and Nef, are known to exploit this pathway to induce pro-inflammatory cytokine production, thereby enhancing viral replication and perpetuating chronic inflammation [94], [95], [96]. The present study suggests that miRNAs further modulate this pathway, amplifying its dysregulation. Similarly, central signaling pathways like PI3K-Akt and mTOR, which are well-documented targets of HIV-1 manipulation, were predicted to be influenced by miRNA dysregulation in this study [97], [98]. Analysis also enriched the pathways involved in apoptosis, such as the p53 signaling axis, underscoring the role of miRNAs in determining infected cell survival. Additionally, the study highlights that neuroinflammation pathways, already known to be dysregulated and contribute to HIV-associated neurocognitive disorders through inflammatory damage in the central nervous system, are also influenced by miRNAs, as revealed by the current analysis [99], [100]. Adding to this, the analysis also enriched the Alzheimer’s disease pathway, underscoring the possible role of miRNA in disease progression. The analysis also revealed the B cell receptor (BCR) signaling dysregulation during HIV-1 infection of MDM, which may have profound significance, as it contributes to immune dysfunction and impaired humoral responses. Dysregulated BCR signaling leads to hyperactivation of B cells, which, paradoxically, results in their functional exhaustion and loss of antigen-specific responses. This impairs the generation of effective antibodies against HIV-1, enabling viral persistence and immune evasion. Moreover, altered BCR signaling disrupts normal B cell differentiation, skewing the balance toward short-lived plasma cells and reducing the generation of long-lived memory B cells, which are critical for sustained immunity. These mechanisms collectively weaken the host's ability to control HIV-1 infection and may contribute to the progression of acquired immunodeficiency syndrome (AIDS) [101]. Apart from the aforementioned pathways, many other pathways were also enriched, highlighting significant molecular dynamics induced by HIV-1 infection. While the dysregulation of several of these pathways has been previously documented, a comprehensive understanding remains elusive. This study attempted to showcase how these pathways, some already known to be disrupted during HIV-1 infection, may be regulated through miRNA-mediated mechanisms. Such insights may have significant practical implications and reveal additional therapeutic targets that have yet to be explored.

The identified dysregulated miRNAs and affected pathways could serve as valuable biomarkers for early HIV infection diagnosis, disease progression monitoring, and treatment response evaluation[67], [102]. Additionally, targeted interventions using miRNA mimics or inhibitors present promising new avenues for HIV cure research, particularly for modulating viral latency and manipulating macrophage reservoirs[103]. However, translating miRNA-based therapies to clinical applications faces substantial hurdles, most notably developing delivery systems that can effectively and safely target infected macrophages while avoiding off-target effects elsewhere in the body [104], [105]. Additional challenges include the inherent instability of miRNAs, as they are rapidly degraded by nucleases in biological fluids, necessitating the use of advanced carriers such as lipid-based or inorganic nanoparticles to protect them during systemic circulation [106], [107]. Furthermore, some delivery vehicles may provoke unintended immune responses, especially when viral vectors or certain lipid formulations are used, which can lead to systemic inflammation [108], [109]. Achieving precise cell-type specificity is also complex, as macrophage subpopulations exhibit heterogeneous surface markers, making it difficult to ensure that therapeutic miRNAs are delivered exclusively to the intended cells [110]. Finally, dose optimization poses a significant challenge because miRNAs often regulate multiple downstream targets, increasing the risk of off-target effects even with localized delivery [111]. While recent advances such as CRISPR/Cas systems and stimuli-responsive nanocarriers show promise in addressing these issues, scalability and long-term safety remain to be established in clinical settings [112], [113]

While these results offered important insights, critical limitations must be acknowledged. The experimental approach using PMA-differentiated THP-1 macrophages, though widely accepted, represents a simplified model that may not fully capture the complexity of HIV-1 infection in primary cells or *in vivo* environments [114], [115]. Addressing this limitation requires further research to validate these findings using primary human macrophages and clinical samples. Additionally, it is important to note that the miRNA target interactions identified in this study were based on bioinformatic predictions and validated databases, which, while valuable, may not fully replicate the biological interactions occurring *in vivo*. Therefore, experimental validation of key miRNA-target relationships will be necessary to confirm their functional relevance [116]. Future studies may employ RNA-seq for higher-resolution analysis, and develop functional assays that directly measure how miRNA manipulation affects HIV-1 replication and latency in macrophages. Investigating synergies between miRNA interventions and current antiretroviral therapies or latency-reversing agents would further strengthen translational potential. This comprehensive examination of miRNA dysregulation during HIV-1 infection not only expands understanding of host-pathogen interactions but also lays crucial groundwork for developing next-generation therapeutic strategies targeting persistent viral reservoirs.

## 5 Conclusions

**Fig. 11:**
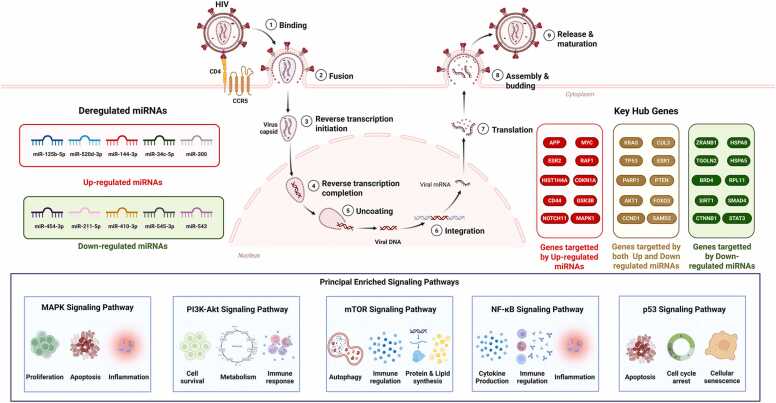
**Overview of HIV-1 infection stages, deregulated miRNAs, targeted hub genes, and principal enriched signaling pathways in macrophages**. The figure illustrates the major stages of the HIV-1 replication cycle in macrophages: (1) Binding of HIV-1 to CD4 and CCR5 receptors, (2) Fusion of viral and host membranes, (3) Initiation of reverse transcription, (4) Completion of reverse transcription, (5) Uncoating of the viral capsid, (6) Integration of viral DNA into the host genome, (7) Translation of viral proteins, (8) Assembly and budding of new viral particles, and (9) Release and maturation of virions. Identified deregulated miRNAs following HIV-1 infection are shown, with upregulated miRNAs indicated in red, and downregulated miRNAs shown in green. Key hub genes targeted by these miRNAs are represented in three groups, comprising genes targeted by upregulated miRNAs (red box), genes targeted by downregulated miRNAs (green box), and genes targeted by both up- and downregulated miRNAs (brown box).The lower panel depicts the principal signaling pathways enriched among the miRNA targets, including the MAPK, PI3K-Akt, mTOR, NF-κB, and p53 signaling pathways, highlighting their major biological roles such as proliferation, apoptosis, immune regulation, cellular metabolism, and inflammation.

The study provides a comprehensive examination of the profound impact of HIV-1 infection on miRNA expression in macrophages, revealing significant dysregulation of both upregulated and downregulated miRNAs. This dysregulation influences key pathways, leading to notable alterations in protein-protein interactions and molecular functions within these cells (Fig. 11). The identified miRNAs are pivotal in regulating essential cellular processes, and their altered expression may play a crucial role in the persistence and pathogenesis of HIV-1. By highlighting the regulatory role of miRNAs in HIV-1 infection, this study offers new insights that could pave the way for the development of targeted RNAi-based therapies and enhance our understanding of viral-host interactions. Further research is needed to validate these findings in clinical settings and explore the therapeutic potential of targeting these miRNA-mediated mechanisms for combating HIV-1 infection and its associated complications.

## Funding

This research was funded by the 10.13039/501100009104Department of Health Research (WSS/2020/000023/AP), India and the Researchers Supporting Project (RSPD2025R1115), King Saud University, Riyadh, Saudi Arabia. The APC was funded by Indian Council of Medical Research and ICMR- National Institute of Translational Virology and AIDS Research, Pune.

## CRediT authorship contribution statement

**Harshithkumar R.**: Writing – original draft, Visualization, Validation, Software, Methodology, Investigation, Formal analysis, Data curation, Conceptualization. **Kaul Mollina**: Methodology, Investigation, Formal analysis. **Chandane-Tak Madhuri**: Methodology, Investigation. **Siddiqi Nikhat J.**: Writing – review & editing, Software, Investigation and Funding acquisition. **Malik Abdul**: Writing – review & editing, Software, Funding acquisition. **Khan Abdul Arif**: Writing – review & editing, Writing – original draft, Visualization, Validation, Supervision, Software, Resources, Project administration, Investigation, Formal analysis, Data curation, Conceptualization. **Mukherjee Anupam**: Writing – review & editing, Writing – original draft, Visualization, Validation, Supervision, Resources, Project administration, Funding acquisition, Formal analysis, Data curation, Conceptualization.

## Declaration of Competing Interest

The authors declare that they have no known competing financial interests or personal relationships that could have appeared to influence the work reported in this paper.

## Data Availability

All data generated or analysed during this study are included in this published article.
